# Comparison of Quality of Life, Anxiety, and Depression Levels in Celiac Patients With Children Without Chronic Illnesses

**DOI:** 10.3390/children12081080

**Published:** 2025-08-17

**Authors:** Erkan Akkuş, Aylin Yücel, Ayhan Bilgiç, Hasan Ali Yüksekkaya

**Affiliations:** 1 Department of Internal Medicine Sciences, Pediatric Gastroenterology, Hepatology, and Nutrition, Health Sciences University Istanbul Başakşehir Çam and Sakura City Hospital, İstanbul 34480, Turkey; 2 Department of Pediatric Gastroenterology, Hepatology and Nutrition, Necmettin Erbakan University Medical Faculty, Meram, Konya 42090, Turkey; aylinyucel@erbakan.edu.tr; 3 Department of Child and Adolescent Mental Health and Diseases, Faculty of Medicine, Izmir University of Economics, İzmir 35330, Turkey; 4 Private Clinic, Altıncaba Plaza, Apt. 401, Kazım Karabekir Street No: 21, Şeyh Sadrettin Neighborhood, Konya 42040, Turkey

**Keywords:** celiac disease, quality of life, mental health, pediatric

## Abstract

**Background:** Celiac disease (CD) is a chronic, immune-mediated condition requiring lifelong adherence to a gluten-free diet. In children, CD can negatively impact not only physical health but also psychological well-being and quality of life. The burden of dietary restrictions, social limitations, and emotional stress may lead to increased anxiety and depressive symptoms. This study aims to compare the quality of life, anxiety, and depression levels in children with celiac disease to those of healthy peers without chronic illness. **Methods:** The research involved a total of 129 individuals aged 8–18 years (64 with celiac disease and 65 healthy volunteers) and their parents. To assess children with celiac disease and healthy children, we used a sociodemographic form that we created, along with the State-Trait Anxiety Inventory (STAI), Trait Anxiety Inventory (TAI), Children’s Depression Inventory (CDI), Pediatric Quality of Life Inventory (PedsQL), and Parent Quality of Life Inventory tests. **Results:** Celiac patients’ diet adherence, parental education level, and family income were found to be significantly associated with quality of life, as well as levels of depression and anxiety. (*p* < 0.037, *p* < 0.04, *p* < 0.004, respectively). Celiac patients had significantly lower BMI SDS (mean −0.55 ± 1.13, *p* < 0.001) and height SDS scores (mean −0.49 ± 1.28, *p* < 0.017). Key factors negatively affecting the quality of life in individuals with celiac disease were difficulty adhering to the diet and low family income levels. **Conclusions:** Elevated anxiety with reduced quality of life highlights the importance of integrating psychosocial support into the routine care of children with celiac disease. A holistic treatment approach that considers the psychosocial well-being of children can significantly improve their quality of life.

## 1. Introduction

Celiac disease is characterized by gluten-related gastrointestinal and extra-gastrointestinal symptoms, the presence of celiac-specific antibodies, HLA-DQ2 or HLA-DQ8 haplotypes, and involvement of the proximal small intestine [1]. In celiac disease, in addition to gastrointestinal symptoms, such as chronic or intermittent constipation, diarrhea, abdominal pain, and bloating, low quality of life, anxiety, and depression, may also occur [1,2].

The ESPGHAN guidelines recommend evaluating children diagnosed with celiac disease using quality of life surveys. This is because a gluten-free diet, especially during the first 6 months, significantly reduces patients’ quality of life [1]. Patients with dyspepsia, mood changes, and depression had a lower mental health composite score compared to others, as they scored poorly in mental health, emotional role, and vitality domains [3]. It has been reported that in families of children with chronic illnesses, the individuals most affected psychologically are the parents and siblings of the sick child [4].

There are studies indicating that celiac disease negatively affects quality of life, particularly impairing social interactions and educational attainment. Dietary restrictions and difficulties faced during the educational process have been identified as the primary factors contributing to this negative impact [5]. Several studies have highlighted the importance of ensuring adequate social and psychological support for patients [5,6].

Celiac disease, a chronic and multisystemic illness, creates anxiety about the future for children and their families and can lead to depressive processes that may affect social life [3]. In this study, we aim to compare the quality of life, anxiety, and depression levels of children and adolescents aged 8–18 who have been diagnosed with celiac disease with those of healthy children and adolescents from the general population who do not have any chronic illnesses. We aim to evaluate the factors influencing quality of life and to assess the mental health status—including anxiety and depression—in pediatric patients with celiac disease by comparing them with healthy peers.

## 2. Methods

### 2.1. Study Group

Our study included 64 children and adolescents aged 8–18 years, along with their parents, who were randomly selected from patients diagnosed with celiac disease and who were followed up between January 2008 and January 2017 at Necmettin Erbakan University according to the ESPGHAN criteria. The control group consisted of 65 healthy volunteers without chronic illnesses, and included their parents. This study is a cross-sectional, comparative study in which pediatric patients with celiac disease were randomly selected from follow-up records and compared to age-matched healthy controls. The necessary ethical approval for this study was obtained from the Medical Research Ethics Committee of Necmettin Erbakan University Meram Medical Faculty (2015/346).

Sociodemographic data forms, as well as the Children’s Depression Inventory, State-Trait Anxiety Inventory, Trait Anxiety Inventory, Pediatric Quality of Life Inventory for ages 8–13, and Pediatric Quality of Life Inventory for ages 13–18, were administered to the children and adolescents in both the patient and control groups. The parents of the participants were also given the appropriate Pediatric Quality of Life Inventory for the children and adolescents’ age groups. The Turkish-validated versions of the questionnaires were administered by Erkan Akkuş, MD, to all participants.

### 2.2. Inclusion and Exclusion Criteria

The study included children and adolescents aged 8–18 years with celiac disease and healthy volunteers without any physical condition that would impair functionality or any chronic illness requiring continuous treatment and follow-up. Informed consent was obtained from all celiac patients and healthy volunteers, as well as from their parents.

### 2.3. Sociodemographic Information Form

This study consists of comparable sociodemographic data of the patients and healthy volunteers included. These data included the name, age, gender, height, weight, body mass index (BMI), and z scores of the patients, according to WHO Anthro ver 3.2.2 and Anthro plus software ver 1.0.4 at the time of diagnosis, as well as the mother’s education level, father’s education level, number of siblings, family income level, and a history of psychiatric illness in the family. In addition to these, children and adolescents in the celiac disease group were asked about their age at diagnosis, how many years they had been diagnosed with celiac disease, diet adherence, objective diet adherence, their difficulty adhering to the diet, comorbid conditions, use of additional medications, and Marsh staging of the small intestine biopsy.

### 2.4. Depression, Anxiety, and Quality of Life Scales

The Children’s Depression Inventory (CDI), developed by Kovacs, is a widely used 27-item self-report scale for assessing depression in children aged 6–17 years [7]. Each item is scored from 0 to 2, with some items reverse-scored. The maximum score is 54, and scores above 19 indicate possible depression.

The State-Trait Anxiety Inventory (STAIC) was developed by Spielberger and colleagues [8] and adapted to Turkish by Öner and LeCompte [9]. The 40-item inventory consists of two scales: State Anxiety (STAI) (measuring the individual’s immediate anxiety) and Trait Anxiety (TAI) (measuring general anxiety levels). Scores range from 1 to 3, with total scores between 20 and 60, where higher scores indicate higher anxiety levels.

The Pediatric Quality of Life Inventory (PedsQL) is a 23-item scale assessing physical and psychosocial health in children [10]. Both children and parents can complete it. Scores range from 0 to 100, with higher scores indicating better quality of life. The scale provides total scale, physical health, and psychosocial health scores. If over 50% of items are unanswered, the scale is invalid.

### 2.5. Statistics

SPSS version 27 was used for the analysis of the study. Harman’s Single Factor Test was applied in this study to assess and rule out the presence of common method bias. Continuous variables are presented with mean, median, standard deviation, maximum, and minimum values. The normality of the data was evaluated using the Kolmogorov–Smirnov and Shapiro–Wilk tests before selecting appropriate statistical tests for analysis. For continuous variables showing normal distribution, comparisons between two groups were made using the independent samples *t*-test, which is a parametric test. For variables not showing normal distribution, the Mann–Whitney U test was used for group comparisons.

The relationship between continuous variables was assessed through correlation analysis, and the relationship was examined using Pearson and Spearman correlation coefficients. A significance level of 95% (*p* < 0.05) was accepted in the study.

## 3. Results

In our study, 64 patients aged 8–18 years diagnosed with celiac disease, who were followed up at the Meram Faculty of Medicine Pediatric Gastroenterology Outpatient Clinic, were included, along with a control group of 65 children and adolescents without any chronic diseases. Celiac disease diagnosis in patients was confirmed by small intestine biopsy.

Among the patients included in the study, 45 were female (70.3%) and 19 were male (29.7%). In the control group, there were 37 female (56.9%) and 28 male (43.1%) participants. The mean age of the patient group was 12.39 ± 3.38 years, while the mean age of the control group was 12.75 ± 3.02 years. When the patient and control groups were compared, a statistically significant difference was found in the height SDS (*p*: 0.017) and BMI SDS (*p* < 0.001) values. Celiac patients had significantly lower values.

A statistically significant difference was found when comparing the maternal education levels between the patient and control groups (*p* < 0.001). In the control group, the number of children whose mother was a college graduate was 22, whereas in the celiac patient group, this number was only three. A statistically significant difference was found when comparing the paternal education levels between the patient and control groups (*p* < 0.001). In the control group, the number of children whose father was a college graduate was 30, whereas in the celiac patient group, this number was only seven. The education level of the parents in the control group was found to be higher. In the verbal assessment where the minimum wage was considered a middle-income level, the family income levels of celiac patients were found to be statistically significantly lower (*p* < 0.001). Statistics related to demographic data are presented in Table 1.

The mean age at diagnosis for girls with celiac disease was 8 ± 3.8 years, while for boys, it was 8.6 ± 3.4 years. Considering the follow-up durations with a celiac disease diagnosis, 16.9% (N = 11) of the patients had been followed for less than 1 year, 35.4% (N = 23) for 1–3 years, 16.9% (N = 11) for 3–5 years, and 30.8% (N = 20) for more than 5 years. When the dietary compliance, laboratory remission status, and dietary adherence difficulties of celiac patients were compared by gender, no statistically significant differences were found in any of these aspects. When the histopathological results reported according to the Marsh classification were compared with age and dietary adherence difficulty, no statistically significant differences were found separately.

The average PHTS values calculated from the quality of life scales for parents were compared, and no significant difference was found (*p* = 0.124). However, when the average PsyHTS and TSS values were compared, a significant difference was found (*p* = 0.02, *p* = 0.04). When the total scores of the Trait Anxiety Inventory (TAI) and State Anxiety Inventory (STAI) applied to the patient and control groups were compared, a statistically significant difference was found in the TAI scores (p = 0.023). However, when the Children’s Depression Inventory (CDI) scores were compared between the patient and control groups, no statistically significant difference was found (p = 0.499) (Table 2). When the PedsQL (Pediatric Quality of Life Inventory) scores were compared between the patient and control groups, no statistically significant difference was found. The results are provided in Table 3.

No statistically significant difference was found in the PedsQL results of the patient group and their parents when evaluated by gender. In the patient group, no statistically significant differences were found in the comparisons of the TAI (Trait Anxiety Inventory), STAI (State Anxiety Inventory), and CDI (Children’s Depression Inventory) scores by gender.

In the patient group, quality of life scales were compared with self-reported dietary compliance, and the comparisons of the child/adolescent PsyHTS and TSS scores were found to be statistically significant (*p*_1_ = 0.021, *p*_2_ = 0.037). In the patient group, the administered scales were statistically compared with dietary compliance, and only the CDI (Children’s Depression Inventory) scores showed a statistically significant difference when compared with self-reported dietary compliance (*p* = 0.023) (Table 3).

To better understand the psychosocial impact of parental education, an analysis was performed in the celiac patient group comparing fathers’ educational levels with outcomes from the adult and child quality of life questionnaires (PedsQL), the State-Trait Anxiety Inventories (STAI and TAI), and the Children’s Depression Inventory (CDI). The results indicated a statistically significant difference in the CDI scores, with the children of fathers with lower educational attainment reporting higher levels of depressive symptoms (*p* = 0.02). In the patient group, the PedsQL scores of children and adolescents were compared based on the paternal educational levels. Statistically significant differences were observed in both the child/adolescent physical health summary score (PHTS) and the child/adolescent total scale score (TSS), with lower scores associated with lower paternal educational levels (*p*_1_ = 0.044, *p*_2_ = 0.035). In the patient group, the parental PedsQL scores were compared according to the fathers’ educational levels. Statistically significant differences were found in both the parental physical health summary score (PHTS) and the parental total scale score (TSS), with lower scores observed in families where the father had a lower educational level (*p*_1_ = 0.021, *p*_2_ = 0.047). An analysis was conducted within the patient group to examine the relationship between maternal educational levels and health-related quality of life outcomes in children and adolescents with celiac disease. The comparison revealed statistically significant differences in both the child/adolescent physical health summary score (PHTS) and the child/adolescent total scale score (TTS), with lower scores observed among children whose mothers had lower levels of education (*p*_1_ = 0.037, *p*_2_ = 0.040). In the patient group, maternal educational levels were compared with the results of the State-STAI, TAI, and CDI. A statistically significant difference was found in the Trait Anxiety Inventory scores, with higher anxiety levels observed in children whose mothers had lower educational levels (*p* = 0.020). These statistical data are presented in Table 4.

To investigate the potential relationship between family income and various psychosocial factors, the celiac patient group’s family monthly income was compared with the PedsQL scores, the State and Trait Anxiety Inventory (STAI, TAI), and the Children’s Depression Inventory (CDI). In the patient group, family income levels were compared with the PedsQL scores of children and adolescents. Statistically significant differences were found in the child/adolescent PHTS, child/adolescent PsyHTS, and child/adolescent TTS scores (*p*_1_ = 0.008, *p*_2_ = 0.018, *p*_3_ = 0.004). In the patient group, the family income levels were compared with the PedsQL scores of parents. Statistically significant differences were found in the parent PHTS and parent TTS scores (*p*_1_ = 0.008, *p*_2_ = 0.012). Family income levels were compared with the results of the TAI, STAI, and CDI scales. A statistically significant difference was found in the STAI results (*p* = 0.021). These findings are summarized in Table 4.

There was no significant difference in the evaluation of previously diagnosed or treated psychiatric disorders. In the patient group, one individual was receiving treatment for major depression and another for bipolar disorder.

The internal consistency of the Pediatric Quality of Life Inventory was assessed using Cronbach’s alpha, yielding a value of 0.87, reflecting a high level of reliability. The internal consistency of the Trait Anxiety Inventory was evaluated using Cronbach’s alpha, which was calculated as 0.84, reflecting a high level of reliability. The Cronbach’s alpha value for the State Anxiety Inventory was calculated as 0.738, indicating acceptable internal consistency. The Cronbach’s alpha value for the Children’s Depression Inventory was calculated as 0.669, which is considered to reflect acceptable internal consistency.

## 4. Discussion

In this study, the PedsQL, STAI, TAI, and CDI scores of children with celiac disease were compared with those of children without chronic illness and not undergoing treatment for any acute condition, along with demographic variables. When the anthropometric measurements were evaluated, the BMI SDS and height SDS values were found to be significantly lower in the celiac group (*p*_1_ < 0.001, *p*_2_ = 0.017). In the celiac group, maternal and paternal education levels, as well as the average monthly income level, were significantly lower (*p*_1_ < 0.001, *p*_2_ < 0.001, *p*_3_ < 0.001). As indicated in many previous studies in the literature, short stature and low BMI values have been observed in celiac patients, and this has been attributed to malnutrition secondary to villous atrophy [1,11,12,13,14]. The necessity of investigating celiac disease as an etiological factor in cases of growth retardation and failure to gain weight has also been emphasized in the ESPGHAN guidelines [1].

According to the results of our study, although the quality of life scale scores were found to be lower in children diagnosed with celiac disease compared to the control group, this difference was not statistically significant. However, when the quality of life scales administered to parents were evaluated, significantly lower scores were observed in the Psychosocial Health Summary Score (PsyHTS) and the Total Scale Score (TTS) (*p*_1_ = 0.02, *p*_2_ = 0.04). These findings suggest that the disease may affect not only the children but also the parents’ perception of quality of life. Previous studies have also demonstrated that quality of life scores in celiac patients are significantly lower, often associated with pre-existing psychopathologies [15,16]. In our study, despite the low prevalence of psychiatric disorders, the particularly low psychosocial scores suggest that this may be due to impaired social interaction stemming from the burden of the disease. This issue has also been addressed in other studies [17,18]. Although the total score (TTS) of the Pediatric Quality of Life Inventory completed by parents was close to the threshold of statistical significance in our study, we believe this finding remains valuable. The economic burden of a gluten-free diet suitable for celiac patients and the difficulties children face in adhering to the diet in social settings among their peers contribute meaningfully to this result.

In our study, when comparing the celiac group with the control group, no statistically significant differences were found in the State Anxiety Inventory (STAI) and Children’s Depression Inventory (CDI) scores. However, the Trait Anxiety Inventory (TAI) scores were significantly higher in the celiac group (*p* = 0.023). This suggests that while situational anxiety and depressive symptoms may not differ markedly between groups, children with celiac disease may have a higher predisposition to experience anxiety as a stable personality trait. The chronic nature of the disease, lifelong adherence to a strict gluten-free diet, and its impact on daily routines and social interactions may contribute to this elevated trait of anxiety [12,18,19,20,21]. These findings underscore the importance of long-term psychological monitoring and support in children diagnosed with celiac disease.

In our study, when dietary compliance was taken into consideration, an increase in psychosocial health summary scores (PsyHTS) and total scale scores (TSS) on the PedsQL was found to be statistically significant among children and adolescents in the celiac group (*p*_1_ = 0.021, *p*_2_ = 0.037). This finding suggests that better adherence to a gluten-free diet is associated with improved quality of life, particularly in psychosocial domains. High levels of dietary compliance may contribute to reduced symptom burden, enhanced social functioning, and overall better daily functioning. As noted in previous studies, these results emphasize the importance of supporting dietary adherence in pediatric celiac patients not only for physical health but also for improvements in psychological and social well-being [15,17,18,22]. Although the association between decreased dietary adherence and reduced psychosocial and overall quality of life in children with celiac disease was statistically significant, the results were close to the *p* < 0.05 threshold. However, we believe this finding is clinically meaningful. As dietary adherence decreases, disease-related symptoms, such as abdominal pain, diarrhea, or constipation, tend to become more pronounced. Moreover, the psychological burden of striving to adhere to a strict gluten-free diet further contributes to increased stress in these patients. These factors support the importance of the observed relationship.

In our study, both maternal and paternal educational levels were positively associated with higher PedsQL scores in both parent-reported and child/adolescent self-reported assessments. As the educational level of the parents increased, statistically significant improvements were observed in quality of life scores. This suggests that higher parental education may contribute to better disease management, increased health literacy, and greater support for the child’s physical and psychosocial well-being. Educated parents may also be more aware of the importance of dietary adherence, psychological support, and medical follow-up, all of which can positively impact the overall quality of life of children with celiac disease. These findings were also demonstrated in the study by Toluna Oflu et al. [22], where only the maternal education level was examined; however, such variables have not been included in the design of many other studies. In the study conducted by Sevinç et al. [15], no statistically significant difference was found. This highlights the role of educational factors in the management of chronic pediatric conditions. Our recommendation is that supporting the nutritional quality and diversity of celiac patients’ diets should be presented as a public health policy.

Our study found that a lower average monthly household income was significantly associated with decreased PedsQL scores in both parent-reported and child/adolescent-reported assessments. This indicates that a lower socioeconomic status may negatively impact the perceived quality of life in children with celiac disease and their families. Statistical comparisons related to family economic status have been addressed in many studies; however, statistically significant results have not often been obtained [12,15,20,22]. In our study, we found that economic status positively correlated with quality of life scores and the State Anxiety Inventory (STAI) scores. Financial constraints may limit access to gluten-free food options, healthcare services, and psychological support, all of which are essential for effective disease management and maintaining well-being. Additionally, economic hardship may contribute to increased stress within the family, further affecting both the child’s and the caregivers’ quality of life. These findings highlight the need to consider socioeconomic factors when developing comprehensive care plans for pediatric celiac patients.

## 5. Conclusions

In conclusion, this study demonstrated that children with celiac disease have significantly lower quality of life scores, and only anxiety measured by the Trait Anxiety Inventory (TAI) was significantly elevated. However, no statistically significant differences were observed in the depression and State-Trait Anxiety Inventory (STAI) scores. These findings highlight the need for targeted psychosocial support that focuses on anxiety management to improve overall well-being in this population. It also showed that major contributing factors to these outcomes included dietary adherence, parental education level, and the average monthly family income.

As a limitation of this study, we were unable to administer the PedsQL to patients under the age of 8, and, therefore, we could not observe the stress factors and their effects during this developmental period. This study is a cross-sectional, comparative study in which pediatric patients with celiac disease were randomly selected from follow-up records and compared to age-matched healthy controls. A total of 64 celiac patients who expressed a willingness to participate and who provided signed informed consent, together with their families, were included in the study. The healthy control group consisted of children without any chronic illnesses who were selected from those presenting to the hospital for acute outpatient complaints. We also acknowledge the possibility that patients or healthy participants may not have answered the questions accurately due to stress. All patients were under follow-up during this period, and this sample represents one of the largest pediatric cohorts reported in the literature on this topic. In future studies, increasing the sample size and using a randomized, blinded design may help reduce the risk of Type I errors. Unfortunately, we were not able to implement such a design in our current study. To assess for potential common method bias due to the use of self-reported measures, Harman’s Single Factor Test was applied using Principal Axis Factoring. The results indicated that the largest single factor accounted for only 16.8% of the total variance, which is well below the commonly accepted threshold of 50%. This suggests that common method variance is not a significant concern in this dataset, supporting the construct validity and reliability of the findings. A multicenter study design that evaluates patients of all age groups during the follow-up process could yield more comprehensive and effective results.

## Figures and Tables

**Table 1:** Demographic data of the study group.

		Celiac		Control	Total	*p*-Value
**Age (mean ± SD)**		12.39 ± 3.38		12.75 ± 3.02	12.57 ± 3.2	0.53
**Female, N (%)**		45 (70.4%)		37 (56.9%)	82 (63.6%)	0.11
**Diagnosis age (yo), mean ± SD**		Female	Male			0.53
		8.12 ± 3.87	8.77 ± 3.39			
**Follow-up (yo)**		4.28 ± 3.17	3.43 ± 2.56			0.30
**Dietary compliance ** **^+^** **, N (%)**		32 (71.1%)	11 (57.89%)			0.54
**BMI SDS**		−0.55 ± 1.13		0.38 ± 1.26	−0.08 ± 1.28	**<0.001**
**Height SDS**		−0.49 ± 1.28		0.07 ± 1.37	−0.21 ± 1.35	**0.017**
**Siblings, N (mean ± SD)**		2.17 ± 1.17		2.37 ± 0.99	2.27 ± 1.05	0.292
**Maternal Education, N**	Primary School	53		26	79	**<0.001**
	High school	8		17	25	
	College	3		22	25	
**Paternal Education, N**	Primary School	37		16	53	**<0.001**
	High school	20		19	39	
	College	7		30	37	
Family Income *	Low	27		9	36	**<0.001**
	Middle	30		20	50	
	High	7		36	43	

* The minimum wage was considered a middle-income level; ^+^ full compliance.

**Table 2:** Comparison of quality of life, anxiety, and depression levels with dietary compliance.

Self-Reported Dietary Compliance	TAI	STAI	CDI	Child/Adolescent PHTS	Child/Adolescent PsyHTS	Child/Adolescent TSS
	Mean	Mean	Mean	Mean	Mean	Mean
Strict Compliance	33.84	31.14	8.40	80.23	80.85	80.64
Partial Compliance	35.58	32.58	10.58	75.33	70.79	72.37
Poor Compliance	45.00	36.00	26.50	85.94	90.83	89.13
*p*-value	0.108	0.390	**0.023**	0.213	**0.021**	**0.037**

**Table 3:** Comparison of parent and child/adolescent PedsQL scores, State Anxiety Inventory (STAI), Trait Anxiety Inventory (TAI), and Children’s Depression Inventory (CDI) scores in celiac and control groups.

	Group	N	Mean	*p*-Value
Parent PHTS ^1^	Celiac	64	75.44	0.124
	Control	65	82.64	
Parent PsyHTS ^2^	Celiac	64	74.66	**0.02**
	Control	65	81.44	
Parent TSS ^3^	Celiac	64	74.93	**0.04**
	Control	65	82.22	
Child/Adolescent PHTS ^1^	Celiac	64	79.13	0.129
	Control	65	83.03	
Child/Adolescent PsyHTS ^2^	Celiac	64	78.36	0.099
	Control	65	82.69	
Child/Adolescent TSS ^3^	Celiac	64	78.63	0.08
	Control	65	82.68	
STAI	Celiac	64	31.75	0.63
	Control	65	31.22	
TAI	Celiac	64	34.65	**0.023**
	Control	65	32.23	
CDI	Celiac	64	9.63	0.499
	Control	65	10.28	

^1^ PHTS (physical health total score); ^2^ PsyHTS (psychosocial health total score); ^3^ TSS (total scale score).

**Table 4:** Comparison of parental educational levels and family incomes with adult and child quality of life questionnaires, State-Trait Anxiety inventories, and the Children’s Depression Inventory in the celiac patient group.

Parents’ Educational Level		TAI	STAI	CDI	Child/Adolescent PHTS	Child/Adolescent PsyHTS	Child/Adolescent TSS	Parent PHTS	Parent PsyHTS	Parent TSS
		Mean	Mean	Mean	Mean	Mean	Mean	Mean	Mean	Mean
None	Maternal	39	37	9	75.00	56.67	63.04	68.75	68.33	68.48
	Paternal	-	-	-	-	-	-	-	-	-
Primary School	Maternal	34.5	31.64	9.61	78.40	78.01	78.14	73.81	73.09	73.34
	Paternal	35.25	32.40	11	76.87	76.04	76.33	71.64	73.08	72.58
High School	Maternal	40.2	34.2	12.8	75.62	76.67	76.30	80.00	79.67	79.78
	Paternal	34.33	34.11	7.17	81.77	80.83	81.16	80.51	73.43	75.90
College	Maternal	26.67	28	5	100.00	95.00	96.74	100.00	97.22	98.19
	Paternal	32	27.14	8.14	85.27	85.24	85.25	84.82	86.67	86.02
*p*-value	Maternal	**0.02**	0.437	0.189	**0.037**	0.082	**0.040**	**0.035**	**0.034**	**0.029**
	Paternal	0.256	0.156	**0.02**	**0.044**	0.072	**0.035**	**0.021**	0.149	**0.047**
Family Income *		TAI	STAI	CDI	Child/Adolescent PHTS	Child/Adolescent PsyHTS	Child/Adolescent TSS	Parent PHTS	Parent PsyHTS	Parent TSS
		mean	mean	mean	mean	mean	mean	mean	mean	mean
Low		35.30	33.56	10.70	76.97	74.63	75.44	70.49	71.54	71.18
Middle		35.26	30.85	9.63	77.55	78.83	78.38	75.96	74.94	75.29
High		29.43	27.00	5.86	94.20	91.19	92.24	92.86	87.38	89.29
*p*-value		0.067	**0.021**	0.073	**0.008**	**0.018**	**0.004**	**0.008**	0.057	**0.012**

* The minimum wage was considered a middle-income level.

## Data Availability

The data generated or analyzed during the study are subject to privacy/ethical/legal restrictions and cannot be made publicly available. However, the data will be provided upon reasonable request to the corresponding author, in compliance with applicable regulations and ethical guidelines.
